# Structure- and Dynamics-Driven Discovery of Small Molecule Inhibitors Targeting a Conserved Pocket near the Fusion Peptide in the Prefusion SARS-CoV‑2 S2 Glycoprotein Subunit

**DOI:** 10.1021/acsomega.6c04313

**Published:** 2026-09-02

**Authors:** Marco Leusciatti, Silvia Rinaldi, Rosaria Arvia, Andrea Catte, Giulia Morra, Simone Giannecchini

**Affiliations:** † National Research Council (CNR), Department of Chemical Sciences and Materials Technologies, 9327 Giulio Natta Institute of Chemical Sciences and Technologies (CNR-SCITEC), Via Mario Bianco, 9, Milan, Milano 20131, Italy; ‡ Department of Chemistry, University of Pavia, Viale Taramelli, 12, Pavia, Pavia 27100, Italy; § National Research Council (CNR), Department of Chemical Sciences and Materials Technologies, 9300 Institute of Chemistry of Organometallic Compounds (CNR-ICCOM), Via Madonna del Piano, 10, Sesto Fiorentino, Firenze 50019, Italy; ∥ Department of Experimental and Clinical Medicine, University of Florence, Florence 50134, Italy

## Abstract

The continuous emergence of severe acute respiratory syndrome coronavirus 2 (SARS-CoV-2) variants has challenged the durability of vaccine-mediated protection, reinforcing the need for broad-spectrum antiviral agents. Herein, we performed a structure- and dynamics-driven virtual screening to identify novel small-molecule inhibitors of SARS-CoV-2 cell entry by targeting a conserved pocket within the S2 subunit of the spike glycoprotein domain. Extensive molecular dynamics simulations of the S2 domain revealed a druggable binding pocket close to the fusion peptide site. Consensus pharmacophore-guided virtual screening of ∼900,000 compounds yielded 11 candidates selected for biological evaluation. The selected compounds reduced SARS-CoV-2 infection in vitro at nanomolar concentrations against different variants with an increased selectivity index. Moreover, viral-cell entry and mechanistic experiments support the selective inhibition of the early steps of viral fusion. Finally, in silico evolutionary analyses showed good conservation of the pharmacophore region among different SARS-related coronaviruses. Our results provide a basis for further optimization studies on novel antifusion SARS-CoV-2 inhibitors with potential cross-clade antiviral activity.

## Graphical abstract

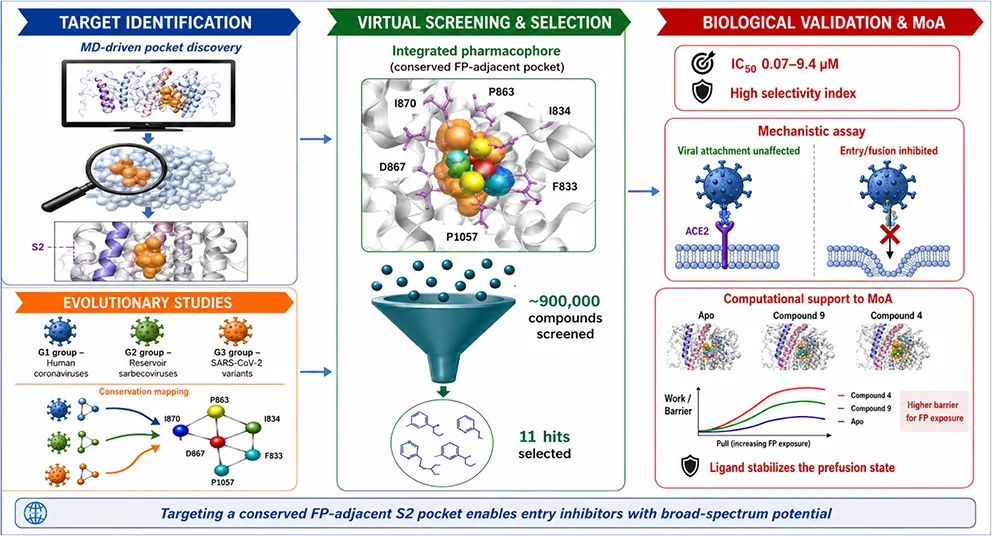

## Introduction

The coronavirus disease 2019 (COVID-19) pandemic, caused by severe acute respiratory syndrome coronavirus 2 (SARS-CoV-2), has highlighted the urgent need for rapidly deployable antiviral interventions.^1,2^ Although vaccines have been pivotal in reducing disease burden,^3^ their protective efficacy has been progressively challenged by viral evolution and the continuous emergence of new variants.^4,5^ The development of new effective antiviral agents therefore remains a key global priority, both for controlling COVID-19 and for preparing against future coronavirus outbreaks.^6,7^ The SARS-CoV-2 spike glycoprotein, like those of HIV and influenza viruses, forms a homotrimer type I transmembrane glycoprotein (class I fusion protein).^8–12^ Each spike protomer is made of two subunits: S1, consisting of the N-terminal domain (NTD), receptor-binding domain (RBD), and two carboxyterminal domains (CTD1 and CTD2),^13^ and S2, which is divided into a fusion peptide (FP), fusion-peptide proximal region (FPPR), internal fusion peptide (FPi, recently recognized as the true fusion peptide), heptad repeat 1 (HR1), central helix (CH), connector domain (CD), heptad repeat 2 (HR2), transmembrane segment (TM), and the cytoplasmic tail (CT).^14^ The spike trimer, after binding to the angiotensin-converting enzyme 2 (ACE2) cellular receptor, undergoes extensive structural changes that drive virus-cell membrane fusion.^14^ In the prefusion state, S1 domains wrap around S2,^15^ with RBDs adopting either receptor-accessible or receptor-inaccessible states.^16–18^ Binding to ACE2 triggers structural rearrangement that promotes S1 dissociation from S2. The subsequent proteolytic cleavage at the 2′ site induces a transition to an elongated conformation that drives to membrane fusion.^8,15,19^ During this process, the fusion peptides of the S2 subunit insert into the host cell membrane, ultimately leading to the postfusion state.^15,20^

Several structural and functional studies have identified key regions of the trimeric spike as potential targets for blocking viral fusion.^21,22^ Additionally, major neutralizing epitopes are located in the receptor-binding domain and the N-terminal domain of the S1 subunit, as well as in the highly conserved S2 subunit domain, which constitutes the core of the fusion machinery.^23,24^

In this context, small molecules targeting the SARS-CoV-2 spike have been explored as modulators of the fusion process,^10,25–27^ offering advantages in terms of oral bioavailability, synthetic accessibility, and potential specificity of action. In silico approaches of the prefusion trimer have further enabled the identification of conserved, druggable regions involved in the fusion transition.^28,29^ Building on this rationale, we focused on a structurally conserved region adjacent to the fusion peptides that becomes exposed upon ACE2 binding and proteolytic activation by the host membrane surface protease Transmembrane Serine Protease 2 (TMPRSS2) or cathepsins in the endosome. This activation induces the exposure of the fusion peptide and the fusion peptide proximal region (FP-FPPR, residues 816-834 and 835-856, respectively). We applied an integrated computational–experimental strategy to identify small molecules targeting this site and to assess their impact on viral entry, mechanism of action, and conservation of the target region across SARS-related coronaviruses.

## Results and Discussion

### Definition of the Target Pocket and Virtual Screening

Guided by molecular dynamics (MD) clustering analysis, the pocket mapping analysis confirmed the presence of a druggable cavity located in close proximity to the protease cleavage site and spatially framed by the FP, in agreement with previous reports (Figure 1).^30,31^ Literature-derived ligands (see representative structures in Supplementary Figure S1)^30,32^ were used as chemical probes in docking experiments to identify recurrent binding modes, delineate key interaction patterns, and refine the pocket definition. Recurrent docking poses revealed a preferential positioning of protonated amine moieties toward a defined electrostatic hotspot within the pocket, indicating that a cationic feature represents a key determinant for productive binding. Residues emerging from recurrent ligand–protein interactions were further clustered to identify the dominant configuration of the pocket. A protein-based pharmacophore model was generated on this structure and merged with a ligand-based model derived from clustering ligand binding poses. The resulting consensus pharmacophore (Figure 1B) consists of eight features. A central positively charged feature defines the primary interaction hotspot, and the distal region displays a branching spatial organization with hydrophobic subpockets. This consensus pharmacophore was used to screen ∼900000 compounds with a formal charge of +1. The screening hits were first filtered according to the consistency between the docking pose and pharmacophore features (see Experimental Section). Then, they were ranked according to their docking scores, and 11 candidates, covering diverse scaffolds, were selected for biological evaluation (Figure 1C and Supplementary Table S1). Structurally, the reference ligands share with our screened series a conserved cationic–aromatic motif, consistent with an electrostatic anchoring point coupled to aromatic packing within the predicted pocket. However, the literature ligands (Supplementary Figure S1 for representative structures and labels) span two main chemotypes: highly lipophilic diaryl amines (CCZ, Fendiline), characterized by extended hydrophobic/aromatic surface with limited hydrogen-bonding capacity, and more compact cationic heteroaryl scaffolds (C2, Vortioxetine), which provide additional directional acceptor/donor features. In addition, the (−)-borneol ester derivate represents a structurally distinct cationic–terpenoid scaffold, defined by a rigid bicyclic hydrophobic framework bearing an ester-linked tertiary amine. Unlike the aromatic-rich chemotypes, this compound relies primarily on a preorganized hydrophobic core combined with a cationic anchor, suggesting an alternative mode of pocket engagement. In contrast, the compounds emerging from our screening campaign display a broader structural heterogeneity. A substantial fraction of the series is characterized by compact cationic heteroaryl frameworks (compounds 3, 8, 10, 11). Alongside these, a subset of ligands adopts more rigid, fused, or bicyclic cationic scaffolds (compounds 4, 5), where the cationic center is integrated within a more conformationally constrained framework. A distinct motif is represented by compounds containing azolyl or azinyl cores linked through thioether functionalities (compounds 7, 9) and amide-bearing aliphatic cationic scaffolds (compounds 1, 6). Finally, one compound stands apart as a highly polar, polyfunctional heteroaromatic scaffold (compound 2). Overall, compared to the literature chemotypes, which are largely dominated by either extended hydrophobic surfaces or well-defined cationic heteroaryl cores, our series explores a wider balance between polarity, conformational flexibility, and scaffold rigidity, pointing to multiple alternative strategies for engaging the same binding pocket. While bulky diaryl scaffolds such as CCZ and Fendiline (Supplementary Figure S2) primarily maximize hydrophobic surface area, and the (−)-borneol ester represents a predominantly hydrophobic terpenoid framework, our data suggest that this pocket may preferentially accommodate geometrically constrained cation–heteroaryl arrangements rather than purely lipophilic or aliphatic hydrophobic architectures.

**Figure 1:**
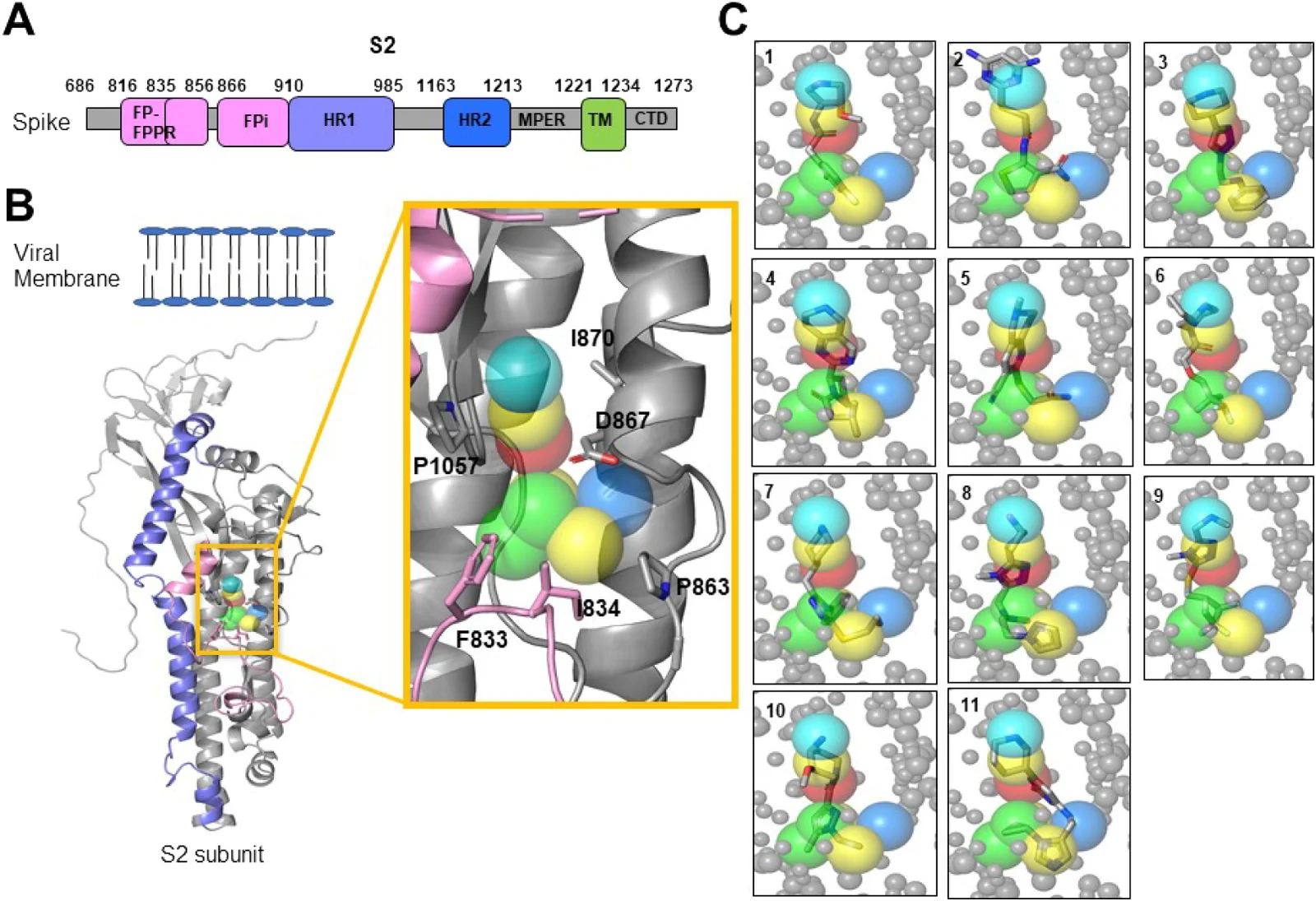
SARS-CoV-2 S2 subunit prefusion target pocket structure, pharmacophore features, and virtual screening of selected compounds. A) Schematic representation of SARS-CoV-2 spike S2 subunit domains. FP, fusion peptide; FPPR, fusion-peptide proximal region; FPi, internal fusion peptide (recently recognized as the true fusion peptide);^14^ HR1, heptad repeat 1; HR2, heptad repeat 2; MPER, membrane proximal region; TM, transmembrane segment; CT, cytoplasmic tail. B) Structure of the dominant conformation of the prefusion spike protomer: Extracted from the trimer MD simulation, this view highlights the pocket region (orange) targeted for ligand binding, adjacent to the fusion peptide (FP) sequence. A close-up of the cavity and pharmacophoric features mapped within the pocket is shown in the orange box. Spheres represent the pharmacophore features with the following color code: yellow: aromatic ring; red: H-bond acceptor; green: hydrophobic; cyan: positive charge; blue: H-bond donor. C) Screening poses of the 11 selected ligands onto the pharmacophore.

### Antiviral Activity of Selected Small Molecule Compounds

The compounds selected from virtual screening were tested against SARS-CoV-2 in Vero E6 cells. The virus and compounds were preincubated for 1 h at 37 °C, at concentrations ranging from 0.1 to 100 μM. Vero E6 cells were infected with SARS-CoV-2 at an MOI of 0.01, and antiviral activity was evaluated by plaque-reduction assay at 72 h postinfection. As reported in Figure 2, all compounds exhibited dose-dependent antiviral activity.

**Figure 2:**
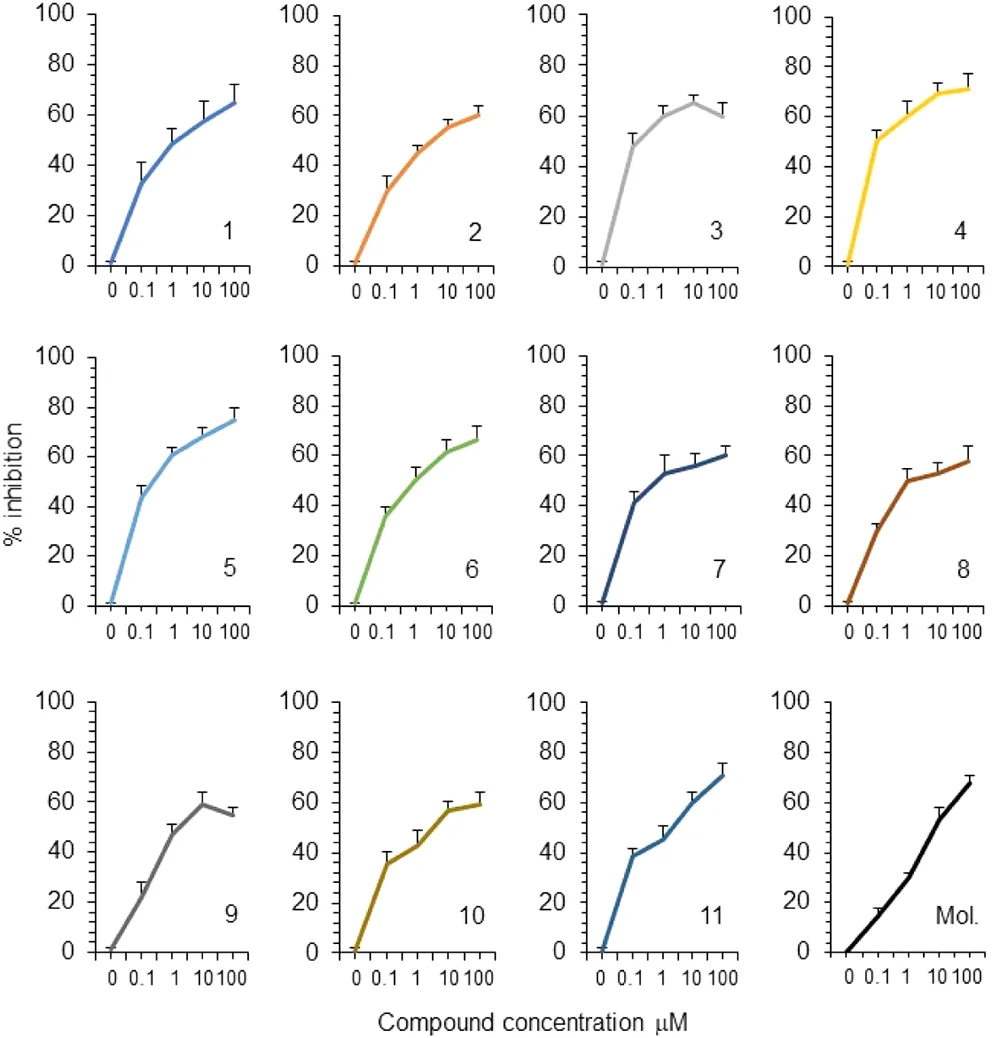
Inhibitory activity of selected small compounds against SARS-CoV-2 Vero E6 cell infection. SARS-CoV-2 infection of Vero E6 cells at a multiplicity of infection (MOI) of 0.01 in the presence of the indicated concentration of compound was assayed with the viral plaque reduction assay. Molnupiravir (Mol) was used as an inhibition control . The values shown are mean ± standard deviation (SD) of 3 independent experiments.

The strongest inhibition profiles were observed for compounds 3, 4, and 5 with activity comparable to molnupiravir, a nucleoside analog used as a positive control, with a submicromolar activity of IC_50_ values ranging from 0.07 to 0.31 μM (Table 1).

**Table 1:**
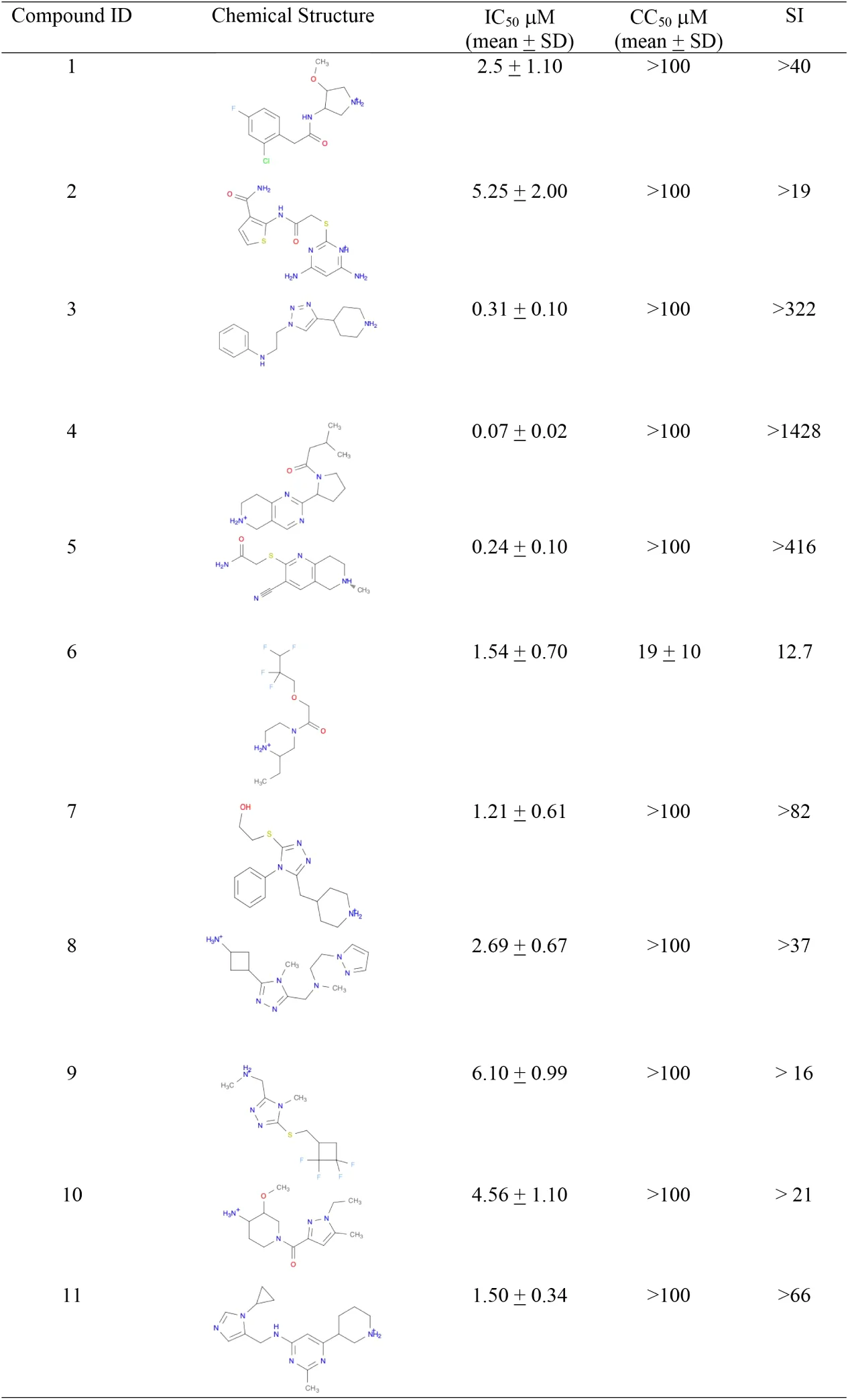
Antiviral Activity and Cytotoxic Effect of the Compounds^a^

a IC_50_, 50% inhibitory concentration; CC_50_, 50% cytotoxicity concentration. Values were obtained in two to three independent assays. SI: selectivity index, calculated as the ratio between CC_50_ and IC_50_.

Based on their antiviral potency and selectivity profiles, compounds 3, 4, 5, and 9 (as compound with the highest IC_50_ activity) were further evaluated against different SARS-CoV-2 variants (Wuhan-like, Delta-like, and Omicron-like variants) obtaining IC_50_ values in the 0.07 to 9.4 μM range (Table 2), confirming cross-variant activity.

**Table 2:** Antiviral Activity of Selected Compounds against Different SARS-CoV-2 Variants^a^,^b^

	IC_50_ μM (mean ± SD)		
Compound ID	Pango lineage B.1	Delta-like pango lineage B.1.617.2	Omicron-like pango lineage BA.1
3	0.41 ± 0.20	0.31 ± 0.15	1.30 ± 0.35
4	0.17 ± 0.02	0.07 ± 0.04	0.27 ± 0.12
5	0.34 ± 0.10	0.28 ± 0.12	0.48 ± 0.12
9	8.10 ± 0.99	6.70 ± 0.63	9.10 ± 0.89

a IC_50_, 50% inhibitory concentration; CC_50_, 50% cytotoxicity concentration.

b Values were obtained in two to three independent assays.

Cytotoxicity was determined in Vero E6 cells by the MTT assay (Figure 3). Interestingly, all compounds were nontoxic up to 100 μM, except for compound 6, which reported a toxic concentration of 6 μM (Table 1). The selectivity index was calculated accordingly, highlighting a promising safety profile for the most active compounds.

**Figure 3:**
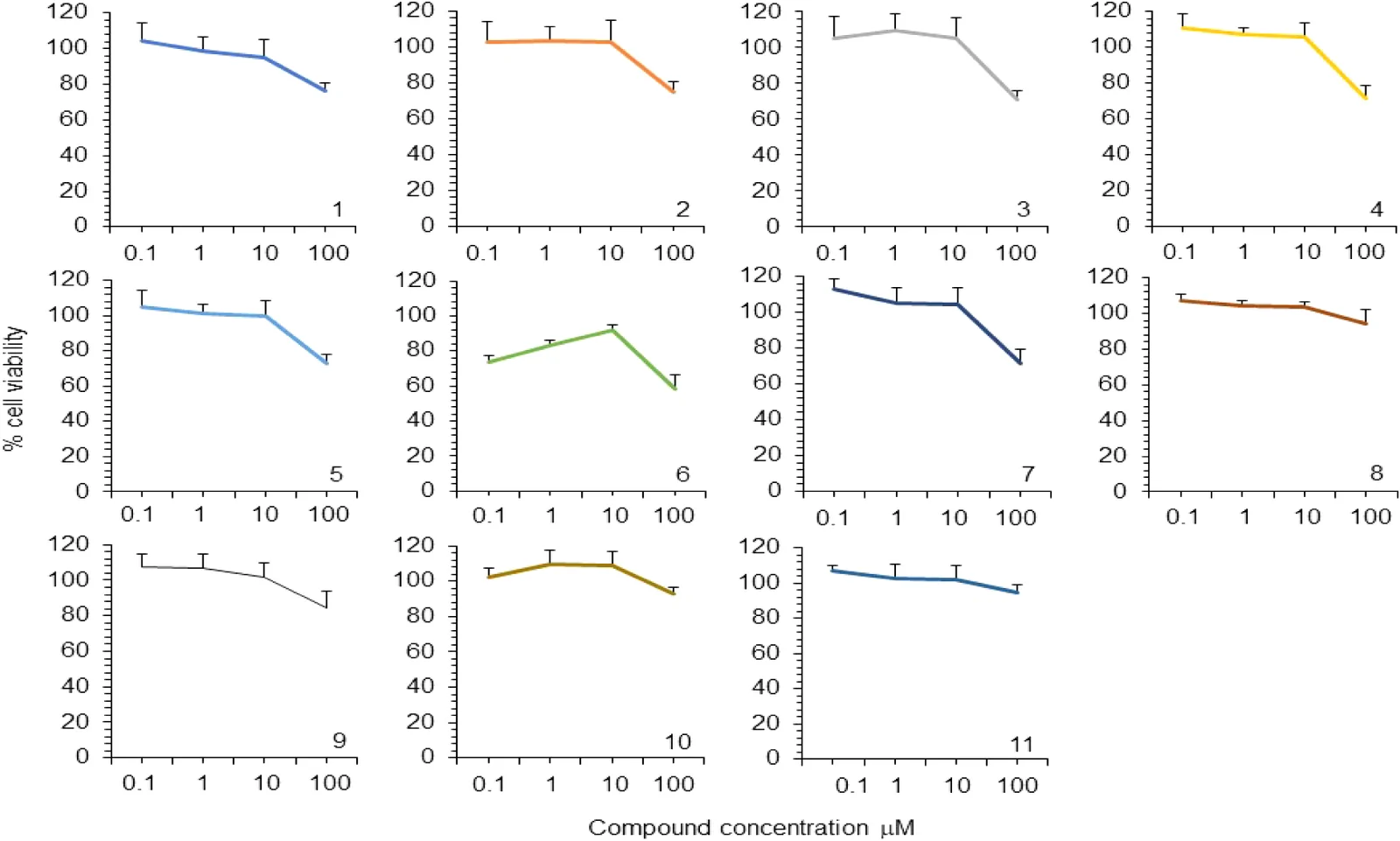
Cytotoxicity of selected small compounds in Vero E6 cells. Cell viability of Vero E6 cells in the same condition in the absence of viral infection was assessed with the MTT assay. The values shown are mean ± standard deviation (SD) of 3 independent experiments.

### Antiviral Cell Entry Activity of Selected Small Compounds

To investigate the mechanism of action, selected lead compounds (compounds 3, 4, 5, and 9) were tested for their ability to block SARS-CoV-2 cell binding or cell entry. To assess whether the compound inhibits virus binding or entry to the cell substrate during infection, after virus-compound mixture incubation with Vero E6 cells at 4 °C or 37 °C, the presence of the SARS-CoV-2 genome was evaluated with a virus-specific real-time PCR standard molecular technique (see Materials and Methods). Thus, following preincubation of the virus and compounds (1 h at 37 °C), the mixture was added to Vero E6 cells and incubated under two conditions:

(i) incubation for 3 h at 4 °C, allowing virus binding but not entry, and (ii) incubation for 1 h at 4 °C followed by 2 h at 37 °C, permitting both virus cell binding and entry. Vero E6 cells were then washed to remove the virus-compound mixture inoculum, and viral RNA was extracted and quantified. As shown in Figure 4a, performing the experiment at 4 °C, compounds 3, 4, 5, and 9 did not significantly affect viral binding compared to the virus-only control. In contrast, in the experiment performed at 37 °C, all four compounds significantly reduced viral entry. To confirm that the inhibitory activity was direct to the inhibition of fusion, a viral fusion assay was carried out using SARS-CoV-2 labeled with octadecylrhodamine B chloride (R18). The R18 assay principle is that the fluorescence of the system increases due to dilution of the self-quenching dye R18 upon fusion between R18-labeled virus and unlabeled Vero E6 cell membranes. The experiments with R18-labeled SARS-CoV-2 were carried out exactly in the same conditions described for those used to measure relative viral RNA levels. As reported in Figure 4b, virus cell incubation at 4 °C did not result in fusion activity. Conversely, virus cell incubation at 37 °C reported a positive fusion activity in the absence of inhibitory compounds. Additionally, virus cell incubation at 37 °C confirmed that compounds 3, 4, 5, and 9 reduced viral cell fusion with activity comparable to an S2-derived peptide PN19 used as a positive control of viral-fusion inhibition.

**Figure 4:**
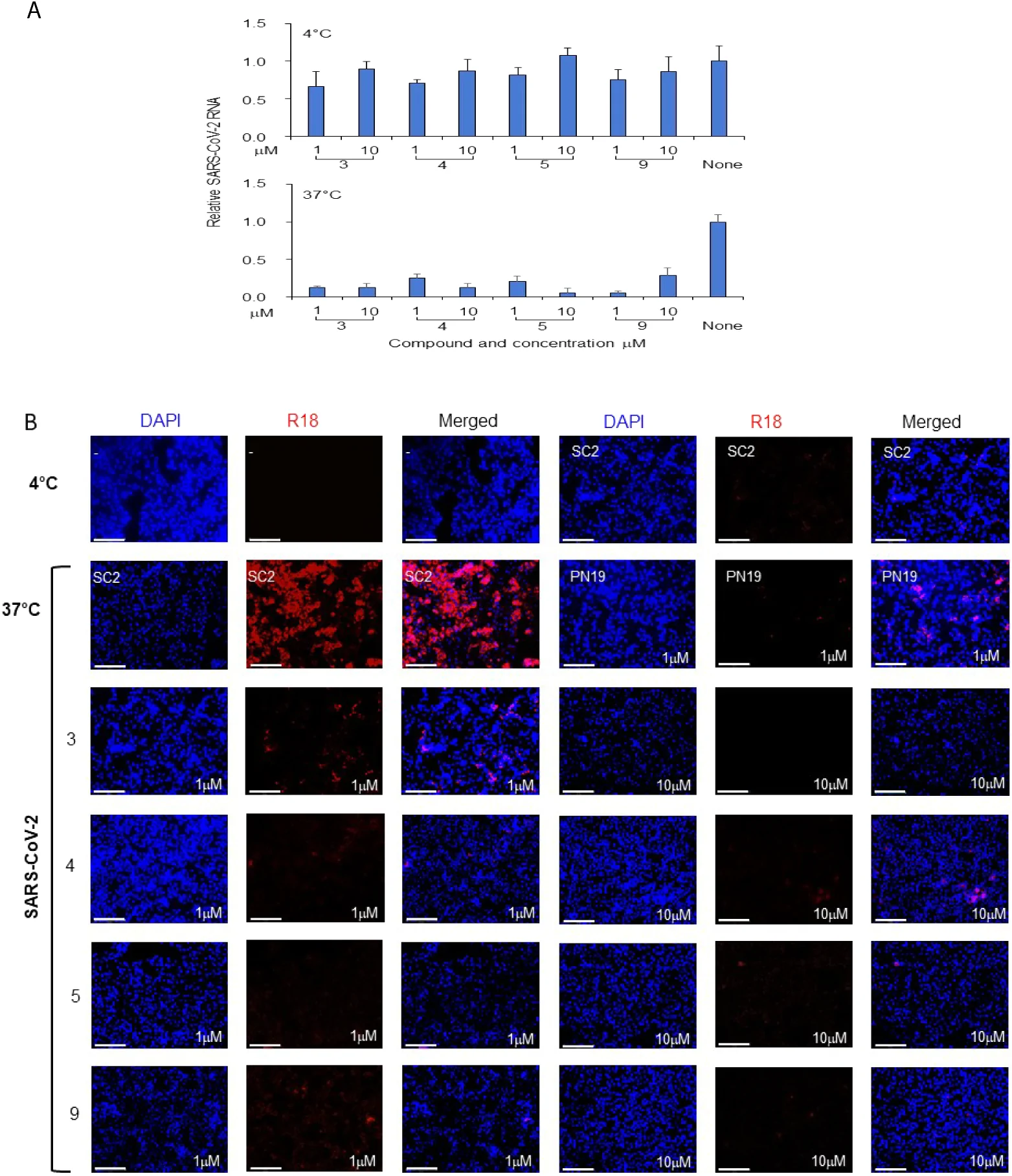
Inhibition of SARS-CoV-2 binding and cell entry. The inhibitory activity of binding and entry of selected compounds was investigated by performing the experiment in the presence of compounds at 1 and 10 μM concentrations added 1 h before infection and incubated at 37 °C with 5% CO2. A) To assess whether the compounds inhibit virus binding or entry to the cell substrate during infection, the virus compound mixture was incubated with Vero E6 cells and, after 3 h at 4 °C (to permit virus adsorption but not entry) or at 4 °C for 1 h followed by 2 h at 37 °C (to permit virus adsorption but not entry). Then, the indicated material, SARS-CoV-2 adsorbed to Vero E6 cells at 4 °C or at 37 °C, was centrifuged at 600 × g for 15 min, and the presence of the SARS-CoV-2 genome was evaluated with viral-specific real-time PCR standard molecular technique. Relative SARS-CoV-2 RNA level inhibitory values were obtained comparing Ct values in the presence of compounds to those obtained with the virus control (None) in the absence of the compounds. The values shown are mean ± standard deviation (SD) of 3 independent experiments. B) SARS-CoV-2 R18-labeled, gradient-purified virus (SC2), after preincubated with selected compounds (1 and 10 μM) for 1 h at 37 °C with 5% CO_2_, was incubated with Vero E6 cells using the same experimental conditions described above. Virus incubation with the S2 derived Peptide PN19, described in a previous study, was used as control of fusion inhibition.^33^ Vero E6 cell nuclei were stained with 4,6-diamidino-2-phenylindole (DAPI). The images of cells were acquired by the inverted multichannel fluorescence and transmitted-light imaging EVOS M7000 microscope (Invitrogen) at 20 × magnification. The scale bars represent 125 μm. −, Vero E6 cell control alone.

These results indicate that the compounds act at the entry stage rather than at the adsorption step, consistent with a mechanism involving interference with membrane fusion, which is in agreement with the predicted binding site adjacent to the fusion peptide.

### Computational Validation of Active Compounds

A moderate inverse monotonic correlation was observed between the pharmacophore shape-fit score of the screened ligands and IC_50_ values (Spearman ρ = −0.61, n = 11, p = 0.046), indicating that improved shape complementarity is associated with increased inhibitory potency (see Experimental Section and Figure S4). Some of the selected ligands, namely 1, 4, 6, 8, 9, 10, and 11, possess stereocenters. The stereoisomers emerging from the screening are detailed in Table S1. As no information on stereochemistry was provided from the vendors, except for compound 8, for which we modeled the correct stereoisomer, it is reasonable to assume that the purchased compounds might consist of racemic mixtures. Despite their stereochemical features, the tested compounds are not expected to undergo racemization or epimerization under the biological assay conditions due to the absence of nearby functional groups associated with conformational instability or any other apparent sources of stereochemical scrambling. Nevertheless, the lack of stereochemical information introduces some uncertainty in the actual activity of those ligands, under the assumption that the reported IC_50_ values likely represent averaged activities across multiple configurations, potentially attenuating the observed structure–activity relationship. To further probe the mechanistic basis of activity, steered molecular dynamics (SMD) simulations were performed to quantify the mechanical work required to extract the FP from its prefusion environment in apo and ligand-bound systems. FP exposure following S2′ cleavage is a prerequisite for membrane fusion in class I viral fusion proteins. Within this framework, we hypothesized that ligand binding may hinder FP deployment by stabilizing the prefusion conformational basin. Accordingly, the mechanical work required for FP extraction in SMD simulations was employed as a surrogate descriptor of ligand-induced interference with this conformational transition. In the apo system, FP extraction required 23.75 ± 2.55 kcal/mol (SEM). In the presence of the most active compound (compound 4), the required work increased to 35.45 ± 2.71 kcal/mol (+49%), whereas the least active compound (compound 9) yielded an intermediate value of 31.83 ± 4.11 kcal/mol. As shown in Figure 5 (Supplementary Figure S3 for the SMD setup), cumulative work profiles diverge after ∼15–20 ns, with ligand-bound systems consistently exhibiting higher mechanical resistance than the apo system. Importantly, the magnitude of the work increase parallels the experimental potency ranking. Notably, the least active compound dissociated from the binding site within 50 ns of the SMD simulation, while the most active compound remained stably bound throughout. Consistently, higher potency is associated with a well-defined protonated amine acting as an electrostatic anchor, a rigid or semirigid heteroaromatic core aligned with the pharmacophoric axis, limited conformational flexibility, and effective occupation of the hydrophobic subpocket. Together, the pharmacophore-based correlation and the SMD-derived mechanical response provide complementary computational support for our structural and mechanistic hypothesis linking structural complementarity to ligand-dependent modulation of FP extraction.

**Figure 5:**
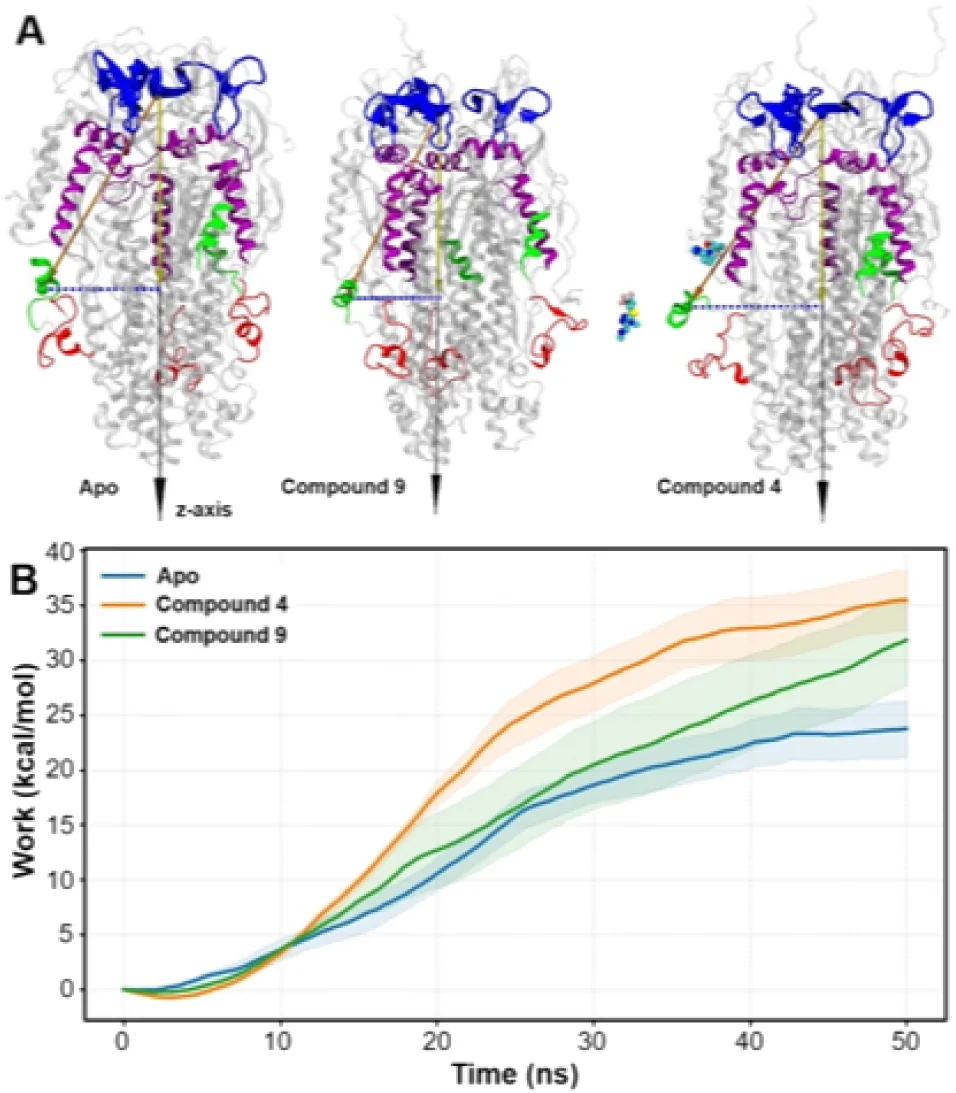
(A) Representative snapshots from 50 ns steered molecular dynamics (SMD) simulations showing constant-velocity extraction of the FP from the prefusion Spike in apo and ligand-bound states. The FP of a single protomer was pulled along the positive *z*-axis away from the S2 core, mimicking exposure following S2′ cleavage of the SARS-CoV-2 spike glycoprotein. The color scheme is consistent with Figure S2. The most active (“best”, compound 4) and least active (“worst”, compound 9) ligands are shown as space-filling models. (B) Mechanical work profiles (mean ± s.e.m.) associated with FP extraction in apo and ligand-bound systems, reported as a function of simulation time.

### Evolutionary Conservation of the Target Binding Site

Finally, we focused on the evolutionary conservation of the S2 domain and, specifically, of the target pocket across selected SARS-CoV-2-related coronaviruses.^34^ Overall, evolutionary profiling of the S2 domain revealed marked functional conservation (mean = 0.876) across three evolutionary groups: G1 (representative human β-coronaviruses), G2 (reservoir sarbecoviruses), and G3 (SARS-CoV-2 variants). Variability was only modestly higher in G1 (0.781) compared to G2 (0.849) and G3 (1.000), indicating that the structural constraints governing S2 are largely preserved even across more distantly related human β-coronaviruses. To determine whether the pocket and pharmacophore features identified in the virtual screening workflow correspond to evolutionarily constrained structural determinants, the S2 residues forming the cavity were mapped in 3D and assigned an evolutionary weight (ef_weight) integrating functional-class conservation and cross-group support. This approach projects sequence conservation into a spatial representation of evolutionary constraint within the pocket. The distribution of ef_weight values indicates that the pocket corresponds to a highly conserved region of S2. The mean ef_weight is 0.850, and 28.6% (20/70) of residues reach ef_weight ≥ 0.9, forming a high-weight subset within the upper tail of the pocket-specific distribution. These positions are virtually invariant in G2 and G3 and remain highly conserved in G1 (support_G2 = 1.000; support_G3 = 1.000; support_G1 = 0.977), demonstrating preservation across divergent β-coronavirus lineages.

At the level of the full 70-residue cavity set, functional-class conservation remained substantial across groups (G1 = 0.745; G2 = 0.804; G3 = 1.000), indicating that the pocket-forming region is not only traceable but also retains a conserved physicochemical profile despite increasing evolutionary distance. Consistently, 72.9% of pocket residues are invariant or display ≤2 variants, and >90% of substitutions at high-weight sites are conservative. Nonconservative substitutions are mainly observed in the more divergent G1/G2 comparisons and are preferentially accommodated at less evolutionarily constrained cavity positions, whereas the highest-weight determinants of the cavity are retained. Together, these findings establish the virtual screening cavity as an evolutionarily constrained structural module central to fusion mechanics and support the presence of a corresponding pocket-forming region across the selected coronavirus panel, extending the potential relevance of this target site beyond SARS-CoV-2.

Comparison between the ligand-derived pharmacophore (DF) and the evolutionary pharmacophore (EF) reveals a structured correspondence. Hydrophobic and aromatic DF elements coincide with the high-weight hydrophobic core centered on PHE833 and ILE834 and extend toward ILE870, while the DF cationic feature lies within ∼5–6 Å of the conserved acidic region (GLU819, ASP820, ASP867), with closest spatial proximity to ASP867, indicating geometric compatibility with the pocket’s conserved electrostatic framework (Figure 6).

**Figure 6:**
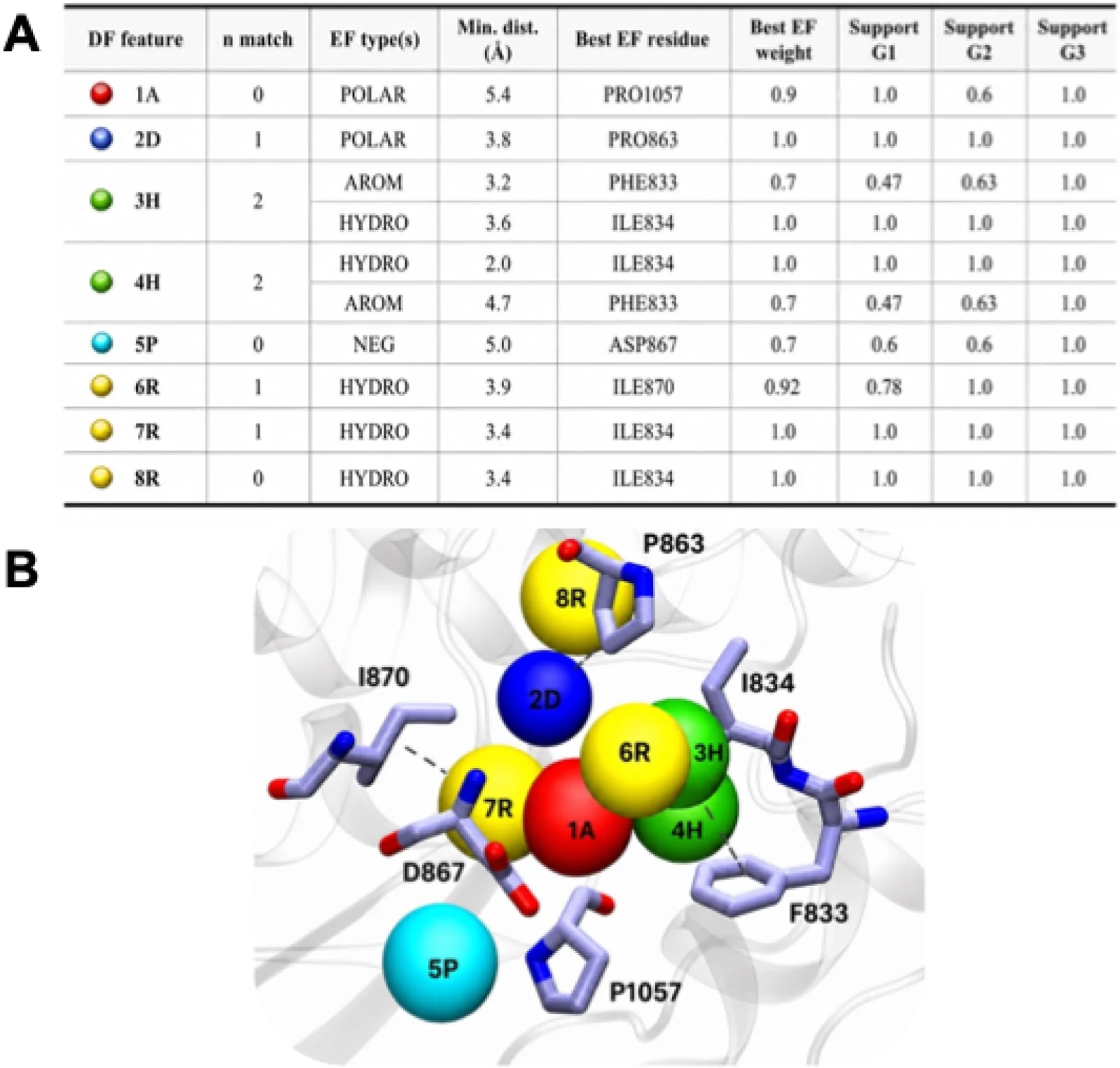
Integrated comparison of the consensus ligand-based pharmacophore (DF) and the evolutionary pharmacophore (EF). (A) Summary table of DF features and their EF correspondence. Feature ID reports feature type (A, hydrogen-bond acceptor; D, hydrogen-bond donor; H, hydrophobic; P, negative ionizable; R, aromatic ring) and sequential index in the consensus model. The n match indicates the number of EF nodes matching each DF feature by pharmacophoric compatibility and distance thresholds. EF type(s) reports the physicochemical class of matched EF nodes (POLAR, AROM, HYDRO, NEG). Min. dist. is the minimum Euclidean distance (Å) between the DF centroid and the nearest EF node. Best EF residue identifies the S2 residue associated with the highest-weight EF match, and Best EF weight reports its pooled evolutionary score, integrating sequence coverage and physicochemical conservation across all groups. (B) Three-dimensional overlay of DF and EF models on the prefusion SARS-CoV-2 S2 domain. DF features are shown as solid spheres, whereas EF-matched residues are displayed in licorice representation, highlighting convergence between ligand-derived and evolutionarily conserved recognition determinants.

Importantly, DF features preferentially align with EF nodes belonging to the high-weight subset, indicating that the pharmacophore geometry captures key evolutionarily constrained determinants of the pocket. This alignment suggests that the DF model is not merely geometrically compatible with the cavity but selectively engages residues under strong evolutionary pressure, linking ligand design to conserved structural mechanics. This selective engagement has direct functional consequences. The most active compound (“best”) preferentially engages EF nodes with a mean ef_weight ≈ 0.94 at DF–EF distances of 2.0–3.3 Å and positions its cationic moiety within ∼3–4 Å of the acidic cluster, enabling electrostatic stabilization and potential cation−π coupling with PHE833. In contrast, the least active analog (“worst”) interacts with residues of lower mean evolutionary weight (≈0.80) and displays increased DF–EF distances to the acidic cluster (>4–5 Å), weakening effective coupling to the conserved electrostatic network. Thus, antiviral potency correlates with selective engagement of structurally constrained determinants rather than simple pocket occupancy (Figure 6). In line with SMD results, the magnitude of mechanical stabilization scales with the degree of engagement of high-weight EF nodes, establishing a quantitative link between evolutionary constraint, structural anchoring, and an increased energetic barrier to FP disengagement. Ligand binding within the DF cavity stabilizes hydrophobic packing around PHE833–ILE834 while reinforcing electrostatic coupling to the conserved acidic cluster, thereby counteracting the conformational plasticity required for FP exposure and HR1 extension. Because these EF nodes are conserved across G2 and G3 and largely retained in G1, targeting this cavity interferes with fusion-essential mechanics rather than mutable surface features.

Targeting the conserved S2 fusion machinery represents a rational strategy to interfere with SARS-CoV-2 entry independently of receptor-binding domain variability and is therefore less susceptible to antigenic drift that has progressively reduced the efficacy of vaccines and RBD-targeting antibodies.^3–5^ Despite the clinical success of fusion inhibitors in other viral systems, most notably HIV and influenza viruses, small-molecule targeting of the SARS-CoV-2 fusion step remains comparatively underexplored relative to protease and polymerase inhibitors.^5,6,11^

In this study, we identify a structurally conserved and druggable pocket within the S2 domain, located adjacent to the FP region, and show that this site can be targeted by small molecules with submicromolar antiviral activity and cross-variant efficacy. This pocket is predicted to become accessible upon receptor engagement and proteolytic activation by TMPRSS2 at the plasma membrane or by cathepsins in the endosome, placing it in a strategic position to influence fusion-associated conformational transitions.^8,15^ The structure–activity relationships emerging from this data set are consistent with the proposed pharmacophore model. Active compounds predominantly adopt compact, geometrically constrained cationic–heteroaromatic scaffolds, in which a protonated amine acts as an electrostatic anchor, while a rigid heteroaromatic core aligns along the pharmacophoric axis and engages the hydrophobic subpocket. Compound 4 represents the most optimized embodiment of this architecture (IC_50_ = 0.07 μM), while similarly potent analogs maintain reduced conformational flexibility and short spatial separation between pharmacophoric features. In contrast, less active compounds display increased conformational freedom, consistent with reduced entropic favorability and suboptimal shape complementarity within the pocket. Cell-viral binding experiments indicate that the active compounds selectively inhibit viral entry without affecting adsorption, supporting an action at the membrane fusion stage rather than at the receptor-binding step. This is consistent with the SMD results, which show that ligand binding increases the mechanical work required for fusion peptide extraction, supporting a model in which ligand binding stabilizes the prefusion conformational basin and raises the energetic barrier associated with fusion-relevant structural transitions. Consistently, compounds displaying higher antiviral potency preferentially engage pharmacophoric features aligned with structurally constrained regions of the pocket, exhibit shorter distances to the conserved acidic cluster, and maintain stable binding during SMD simulations. This selective engagement correlates with increased mechanical resistance to fusion peptide extraction, indicating that antiviral activity arises from interaction with structurally constrained determinants rather than nonspecific pocket occupancy. A distinctive feature of this work is the evolutionary validation of the target site. The convergence between the ligand-derived pharmacophore (DF) and the evolutionarily constrained residues of the cavity (EF) indicates that the interaction points required for ligand activity correspond to positions under strong selective pressure across divergent SARS-related coronaviruses. This supports the functional relevance of the identified pocket and suggests that compounds engaging high-weight EF nodes may be intrinsically more robust to adaptive escape as mutations at these positions would likely compromise fusion mechanics. The identified pocket and its associated pharmacophore are consistent with previously reported small-molecule binding regions within the S2 subunit, including cavities adjacent to the fusion peptide that can accommodate cationic–aromatic chemotypes.^28,29^ Our results extend these observations by integrating structural, mechanistic, and evolutionary evidence. Notably, the structural and mechanistic evolutionary conservation of the identified S2 pocket site makes it unlikely to develop antiviral escape mutants although this remains to be experimentally proven. Several limitations should be mentioned. The biological validation is currently limited to Vero E6 cells, and further studies in additional physiological systems are required. The SMD analysis, while mechanistically informative, is based on a limited subset of compounds and should be extended to a broader chemical series. In addition, stereochemical effects and pharmacokinetic properties remain to be fully characterized and will be critical for future lead optimization and in vivo evaluation.

Most importantly, our model still lacks structural validation. While the overall consistency with the literature ligands and the biological evidence strongly suggests that the actual binding site of the ligands could be in the identified region on the spike protein, both direct binding to the spike protein and physical interaction with the hypothesized pocket remain to be experimentally proven and will be the subject of future work.

## Conclusion

Overall, this work demonstrates that the conserved pocket within the prefusion S2 subunit represents a highly druggable target for SARS-CoV-2 entry inhibition. The alignment between key pharmacophore features and evolutionarily constrained residues underscores both the structural conservation and the functional importance of this site. Moreover, the structural and dynamical modeling gives a consistent picture of the molecular mechanism of action of the ligands. Collectively, these findings establish a robust foundation for the rational design of next-generation fusion inhibitors with a broad spectrum of antiviral potential.

## Experimental Section

### Cells, Viruses, and Chemical Compounds

The cell lines used were Vero E6 (BS-CCLO87, ATCC, Rockville, MD, USA) cultivated in Dulbecco’s Modified Eagle Medium (DMEM, Merck, Germany) supplemented with 10% fetal bovine serum (FBS, Merck, Germany). The viruses used were SARS-CoV-2 clinical isolates (SCV2/Fi/3/22, Pango lineage B.1; SCV2/Fi/2/21, Delta Pango lineage B.1.617.2; and SCV2/Fi/1/22, Omicron Pango lineage BA.1) grown on Vero E6 cells and titrated by the plaque method. The viral stocks, consisting of cell-free supernatants of acutely infected cells, were aliquoted and stored at −80 °C until used. The chemical compounds were obtained from Ambinter c/o Greenpharma SAS (Ambinter c/o Greenpharma SAS, Orlèans, France) (Amb31365959, Amb520928, Amb22310043) and from Mcule (Mcule.com, Budapest, Hungary) (MCULE-2123355862, MCULE-5953273415, MCULE-3835849185, MCULE-1882300118, MCULE-4248285817, MCULE-7635943229, MCULE-9942300320, MCULE-7042633591).

### Protein Modeling

The fully glycosylated S protein head-only model (residues 1–1146) of the Wuhan wild type, based on PDB ID 6VSB, was obtained from the CHARMM-GUI COVID repository.^16^ Starting from the Wuhan sequence (UniProt code P0DTC2), residues were mutated based on the Omicron BA.1 sequence, and the glycosylation was modified accordingly. After removing the S1 domain, the S2 domain, comprising residues 686–1146, was used in this work as the relevant construct that is exposed toward the host membrane, preceding viral fusion and upon cleavage at the S1/S2 furin site. Additionally, the S2′ site at residues 815–816 was modeled as cleaved, mimicking the effect of the host protease TMPRSS2, or cathepsins in the endosome, that induce exposure of the bona fide fusion peptide and the fusion peptide proximal region (FP-FPPR; residues 816–834 and 835–855, respectively). The comparison of the simulation starting conformation with the cryo-EM structure of the Omicron variant (PDB ID 7WEC) confirms the validity of the model with a Ca RMSD of residues 710–1149 of 1.8 Å (Supplementary Figure S1).^35^

### Molecular Dynamics Simulations

The construct was generated, solvated, and ionized using the CHARMM 46b1 academic version, the CHARMM36m force field, and in-house scripts adapted from standard CHARMM-GUI input generator files.^36,37^ A cubic box including TIP3 water molecules was built, and Na+ and Cl– ions were added to reach 0.1 M ionic strength, resulting in 305232 atoms. Minimization, restrained equilibration, and unrestrained equilibration/early production were run for 10000 steps, 30 ns, and 100 ns, respectively, using NAMD 3.0.^38^ Production runs were subsequently carried out using OpenMM 8, starting from the NAMD 3.0 final structure.^39^ First, 6 replicas of 1.1 μs were run. Subsequently, each replica’s ending structure was used to start 6 more replicas, accumulating a total simulation time of ∼25 μs. The 25 μs data set was used for the analysis.

### Pocket and Pharmacophore Definition, Virtual Screening, and Hit Validation

To generate target structures for the in silico drug discovery approach, a metatrajectory was first built by dissecting the molecular dynamics (MD) trajectory into three monomer trajectories, which were concatenated yielding 20,569 frames. The metatrajectory was subjected to a clustering procedure focusing on the C_α_ atoms using GROMACS 2024 with the GROMOS algorithm and a cutoff of 0.3 nm.^32^ A set of 77 ligands collected from the literature was prepared using LigPrep, and only the protonation state corresponding to a + 1 net charge was retained for subsequent calculations.^30,31,40^ Docking poses were generated using Glide, under standard SP settings, and subsequently clustered to identify recurrent binding modes and key interaction patterns and refine the pocket definition.^41^ The resulting selected pocket residues (SARS-CoV-2 S2 spike amino acid residues 729, 730, 731, 732, 733, 734, 774, 775, 777, 778, 816, 819, 820, 822, 823, 828, 829, 832, 833, 834, 835, 837, 860, 861, 862, 863, 864, 865, 866, 867, 868, 870, 871, 952, 955, 956, 1055, 1056, 1057, 1058, 1059) were subjected to a second clustering analysis with a cutoff of 0.3 nm. The dominant pocket-focused cluster (72% of the ensemble) was subjected to a second round of docking calculations using the same chemical probes to confirm the persistence of the identified binding site, yielding the final target model. The target-based pharmacophore model was generated with Phase using the automated pharmacophore detection protocol while retaining excluded volume constraints.^42^ The ligand-based pharmacophore model was constructed from the clustered ligand binding poses using target-ligand complex settings (Supplementary Figure S2). Merging the two models led to the final consensus pharmacophore used for screening. To define the screening library, compounds were downloaded from the ZINC database as predefined tranches, selecting in-stock molecules with available 3D structures, +1 formal charge, and lead-like properties.^43^ This search yielded 913,977 protomers distributed across 320 tranches. Within Phase, a database was generated using standard settings, producing up to 50 conformers per compound, followed by minimization of the output conformers. Pharmacophore-based virtual screening was then performed using the consensus pharmacophore model. Only ligands retaining a + 1 formal charge were considered for further analysis. The top 5000 ranked candidates emerging from the screening were subsequently docked to the pocket using Schrödinger Glide under standard precision (SP) and default settings.^40^ Pose consistency was evaluated by calculating the RMSD between the pharmacophore-aligned pose and the corresponding docking pose, filtering out compounds over 4 A RMSD. The surviving ligands were then ranked according to the docking score. The shape-fit score relative to the consensus pharmacophore model was calculated using Phase. This metric quantifies how well the ligand volume matches the 3D geometry of the pharmacophore hypothesis.

### SARS-CoV-2 Plaque Reduction Assay

The inhibitory activity of small compounds against SARS-CoV-2 variants was assessed in Vero E6 cells in 6-well plates using a multiplicity of infection (MOI) of 0.01. The plaque reduction assay (PRA) of the small compound on viral replication was analyzed at 3 days and compared to the viral control grown in the absence of the compounds. Briefly, virus was mixed with a serial dilution (final concentrations ranging from 0.1 to 100 μM) of the compound prepared in dimethyl sulfoxide (DMSO; Merck, Germany) and incubated for 1 h at 37 °C in humidified air with 5% CO_2_. Virus mixed with DMSO alone was used as control of replication. Then, the viral mixtures were inoculated on Vero E6 cells in the presence of L-1-tosylamido-2-phenylethyl-chloromethyl-ketone-treated trypsin (2 μg/mL; Sigma, St. Louis, MO, USA) to improve viral cell entry^44^ and incubated for 1 h at 37 °C in humidified air with 5% CO_2_. After the cells were washed with PBS 1X, an overlay medium composed of 0.5% Sea Plaque Agarose diluted in propagation medium in the absence of FBS was added to each well. Additionally, L-1-tosylamido-2-phenylethyl-chloromethyl-ketone-treated trypsin (2 μg/mL; Sigma, St. Louis, MO, USA) was added to the monolayer. The PRA of the small compound on viral replication was analyzed at 3 days compared to the viral control grown in the absence of the compounds. The fifty percent inhibitory concentration (IC_50_) was calculated using the predicted exponential growth function in Microsoft Excel.

### Inhibition of SARS-CoV-2 Binding and Cell Entry

The inhibition of binding and entry of selected lead compounds was investigated by performing the experiment in the presence of the compound at 1 and 10 μM concentrations, added 1 h before infection, and incubated at 37 °C with 5% CO_2_. To assess whether the compound inhibits virus binding or entry into the cell substrate during infection, the virus compound mixture was incubated with Vero E6 cells and, after 2 h at 4 °C or at 37 °C, the presence of the SARS-CoV-2 genome was evaluated with a virus-specific real-time PCR standard molecular technique. Briefly, the compound (1 and 10 μM) was preincubated with SARS-CoV-2 for 1 h at 37 °C with 5% CO_2_. Virus mixed with DMSO alone was used as control of replication. Then, the virus compound mixture was incubated with Vero E6 cells (5 × 10^5^) at 4 °C (to permit virus adsorption but not entry) or at 37 °C (to permit both virus adsorption and entry) in the presence of L-1-tosylamido-2-phenylethyl-chloromethyl-ketone-treated trypsin (2 μg/mL; Sigma, St. Louis, MO, USA). Then, the indicated material, SARS-CoV-2 adsorbed to Vero E6 cells at 4 °C for 3 h or at 4 °C for 1 h and subsequently at 37 °C for an additional 2 h, was centrifuged at 600 × g for 15 min and assayed by molecular quantification. RNA was extracted from infected cells (1 × 10^5^) with the RNeasy Mini Kit (Qiagen) according to the manufacturer’s instructions. One hundred nanograms of total RNA of each sample were reverse-transcribed and amplified by real-time PCR in a 25-ml PCR reaction mix (AgPath-ID One-Step RT-PCR, Applied Biosystems) using primers targeting the N gene of SARS-CoV-2 (primer forward SC2-For 5′ CTG CAG ATT TGG ATG ATT TCT CC 3′, reverse SC2-Rev 5′ CCT TGT GTG GTC TGC ATG AGT TTA G3′, and probe SC2-Probe FAM-5′ ATT GCA ACA ATC CAT GAG CAG TGC TGA 3′-MGB). The mixture was amplified in a Rotor-Gene Q real-time apparatus (Qiagen), and the reaction was carried out at 95 °C for 10 min followed by 40 cycles (20 s at 95 °C, 60 s at 60 °C; the fluorescence was recorded at 70 °C). All reactions were done in duplicate. The inhibitory activity of virus binding and cell entry was calculated by comparing the SARS-CoV-2 RNA cycle threshold (Ct) levels obtained in the presence of compounds with the SARS-CoV-2 RNA Ct values obtained in the presence of DMSO alone.

An additional viral fusion assay, based on fluorescence dequenching of octadecyl rhodamine (R18)-labeled purified virus (Molecular Probes), was performed.^45^ SARS-CoV-2 virus was labeled with the lipophilic fluorescent dye R18 (2 mM) at 37 °C for 1 h. Then, SARS-CoV-2 R18-labeled gradient-purified virus was preincubated with selected compounds (1 and 10 μM) for 1 h at 37 °C with 5% CO_2_. Virus mixed with DMSO alone was used as control of replication. Then, the virus compound mixture was incubated with Vero E6 cells (5 × 10^5^) at 4 °C for 3 h (to permit virus adsorption but not entry) or for 1 h at 4 °C followed by 2 h at 37 °C (to permit both virus adsorption and entry) in the presence of L-1-tosylamido-2-phenylethyl-chloromethyl-ketone-treated trypsin (2 μg/mL; Sigma, St. Louis, MO, USA). After cells were washed with PBS 1X, the images of cells fixed with paraformaldehyde (4%) were acquired by the inverted multichannel fluorescence and transmitted-light imaging with an EVOS M7000 Imaging System microscope at 20× magnification. The scale bars represent 125 μm.

### Cell Cytotoxicity Assay

The cytotoxicity of compounds was evaluated by the MTT reduction assay. Vero E6 cells were plated at a density of 10^4^ cells per well in a flat-bottom 96-well culture plate and allowed to adhere overnight. When the cell layers were confluent, the medium was removed, the wells were washed twice with PBS, treated with 100 μL of MEM alone or with or without the appropriate concentrations of the compounds (final concentrations ranged from 0.1 to 100 μM), and incubated at 37 °C in a CO_2_ incubator for 72 h. After treatment, an MTT kit (Roche, Milan, Italy) was used according to the supplier’s instructions, and the absorbance of each well was determined using a microplate spectrophotometer at a wavelength of 595 nm. Cytotoxicity was calculated by dividing the average optical density of the treated samples by the average of the mock-treated samples in the presence of DMSO alone.

### Steered Molecular Dynamics (SMD)

SMD simulations were performed to quantify the mechanical work required to extract the Fusion Peptide (FP) from its prefusion environment. Pulling work was used as a mechanical descriptor of FP extraction. Simulations were conducted in both apo and ligand-bound states (three independent replicas per system) to enable direct comparison of force and work profiles. The apo structure corresponds to the final receptor model employed in the virtual screening workflow (dominant pocket-focused cluster C1). SMD simulations were performed only for the two compounds displaying the best and worst experimental IC_50_ values to evaluate the consistency of the proposed mechanism, i.e., interference with FP deployment (Supplementary Figure S3). For each compound, the docking pose with the highest ligand efficiency was selected. As docking was performed on a monomeric construct, the chosen target–ligand complex was aligned onto the corresponding frame of the initial trimeric MD trajectory, and the full trimer was reconstructed by restoring the remaining protomers from that frame to generate the ligand-bound systems used for SMD. All models were simulated using NAMD 3.0 with the CHARMM36m force field.^37^ Ligand parameters were generated with CHARMM-GUI Ligand Reader and Modeler.^35,36^ Each solvated and ionized trimer was aligned along the *z*-axis, defining the pulling direction. FP residues 816–834 of a single protomer were selected as the pulling group. Systems were energy-minimized (5,000 conjugate gradient steps), heated from 0 to 310 K using a Langevin thermostat, and equilibrated in the NPT ensemble at 310 K and 1 atm prior to SMD production runs. Constant-velocity pulling (0.25 Å ns^–1^) was applied to the center of mass of FP backbone atoms along the positive direction of the *z*-axis, using a harmonic spring constant of 5 kcal mol^–1^ Å^–2^. The center of mass of the C-terminal region (residues 1097–1122 in all three protomers) was restrained (1 kcal mol^–1^ Å^–2^) to provide a mechanical reference frame. Since the pulling was applied along the *z*-axis, the x- and y-components of the pulling force are zero; the mechanical work was therefore estimated as the product of the z-component of the pulling force and the displacement of the FP along the *z*-axis. Pulling forces and work were calculated using in-house Tcl scripts in VMD 1.9.4.^46^

### Evolutionary Conservation

Spike protein sequences were grouped into three predefined SARS-related coronavirus classes: (i) human β-coronaviruses (G1), (ii) nonhuman sarbecovirus reservoir species (G2), and (iii) SARS-CoV-2 variants of concern and interest (G3) (see Supporting Information for GenBank accession number). All analyses were restricted to the S2 subunit (residues 686 to the C-terminus, P0DTC2 numbering). A single multiple sequence alignment (MSA), anchored to the SARS-CoV-2 reference sequence (UniProt P0DTC2), ensured one-to-one correspondence between alignment columns and S2 positions. For each position and evolutionary group, column-wise metrics were computed over nongap residues, including gap fraction, Shannon entropy (amino acid and physicochemical classes), class-based conservation score, hydropathy (Kyte–Doolittle scale), and identity to the reference sequence. A pooled profile was obtained by averaging conservation and coverage metrics across groups, enabling both group-specific and cross-group analyses. To evaluate whether the ligand-binding pocket corresponds to evolutionarily constrained determinants, conserved S2 residues within the cavity were mapped onto the prefusion Spike structure to define an evolutionary pharmacophore (EF). In this representation, each EF node corresponds to a cavity residue in three-dimensional space and is weighted by evolutionary constraint (ef_weight), integrating sequence coverage and physicochemical conservation. Group-specific weights were retained for intra- and interclade comparisons, while a pooled score defined the primary EF model.

The EF was compared to the ligand-derived pharmacophore (DF) by computing pairwise Euclidean distances between feature centers. Although the DF integrates both ligand-based and receptor-informed information, it remains ligand-centric, as features describe the spatial arrangement of interaction points required for ligand activity. In contrast, the EF provides a target-centric representation of evolutionarily constrained residues within the cavity. Matches were defined based on pharmacophoric compatibility and distance thresholds (5.0 Å for hydrophobic/aromatic features, 4.5 Å for polar/charged features, and 5.0 Å for ring features). This analysis enables a systematic assessment of whether ligand design hypotheses engage receptor positions under evolutionary constraint.

## Supplementary Material



## Notes

Data relative to representative ligands from the literature used for preliminary docking and pocket analysis to guide the pharmacophore definition, selected compounds emerging from the screening, representative snapshots of steered molecular dynamics (SMD) simulations, multiple sequence used for the alignment, and structural superposition of the subset residues 710–1149) reference spike structure (PDB 7WEC) and the modeled Omicron variant are reported in Table S1 and Figures S1–S3 (PDF)

## Abbreviations:

SARS-CoV-2: Severe acute respiratory syndrome coronavirus 2
COVID-19: Coronavirus disease 2019
NTD: N-terminal domain
RBD: receptor-binding domain
CTD1 and 2: carboxyterminal domains 1 and 2
FP: fusion peptide
FPPR: fusion-peptide proximal region
FPi: internal fusion peptide (internal fusion peptide region)
HR1–2: heptad repeat regions 1 and 2
MPER: membrane proximal external region
TM: transmembrane region
CT: cytoplasmic tail
ACE2: angiotensin-converting enzyme 2
TMPRSS2: transmembrane serine protease 2
MD: molecular dynamics
FBS: fetal bovine serum
MOI: multiplicity of infection
PRA: plaque reduction assay
PFU: plaque forming unit
IC50: 50 percent inhibitory concentration
SD: standard deviation
MTT: (3-[4,5-dimethylthiazol-2-yl]-2,5-diphenyl tetrazolium bromide)
SMD: steered molecular dynamics
DF: ligand-derived pharmacophore
EF: evolutionary pharmacophore
DMEM: Dulbecco’s Modified Eagle’s Medium
DMSO: dimethyl sulfoxide
