# Supplementary material for: Structure- and Dynamics-Driven Discovery of Small Molecule Inhibitors Targeting a Conserved Pocket near the Fusion Peptide in the Prefusion SARS-CoV‑2 S2 Glycoprotein Subunit
Source: ACS Omega. 2026 Sep 2;11(37):55486–98. doi: 10.1021/acsomega.6c04313 (PMC13613865; doi:10.1021/acsomega.6c04313)
Supplement: Supplementary file 1 [file ao6c04313_si_001.pdf]

Supplementary Information

Structure and dynamics-driven discovery of small molecule inhibitors targeting a conserved pocket near the fusion peptide in pre-fusion SARS-CoV-2 S2 glycoprotein subunit

Marco Leusciatti[1,2]*, Silvia Rinaldi[3]*, Rosaria Arvia[4], Andrea Catte[3], Giulia Morra[1]#, Simone Giannecchini[4]#

1- Department of Chemical Sciences and Materials Technologies, Giulio Natta Institute of Chemical Sciences and Technologies, National Research Council (CNR), Milano, Lombardy, Italy

2- Department of Chemistry, University of Pavia Viale Taramelli 12 27100 - Pavia Italy

3- CNR - Istituto di Chimica dei Composti Organometallici (CNR-ICCOM), Via Madonna del Piano 10, Sesto Fiorentino I-50019, Italy.

.4- Department of Experimental and Clinical Medicine, University of Florence, 50134 Florence, Italy

*These authors contributed equally

#Correspondence should be addressed to: Dipartimento di Medicina Sperimentale e Clinica, Università di Firenze, Viale Morgagni 48, I-50134 Firenze, Italy; simone.giannecchini@unifi.it; National Research Council (CNR) Department of Chemical Sciences and Materials Technologies, Giulio Natta Institute of Chemical Sciences and Technologies, , Milano, Lombardy, Italy; giulia.morra@scitec.cnr.it

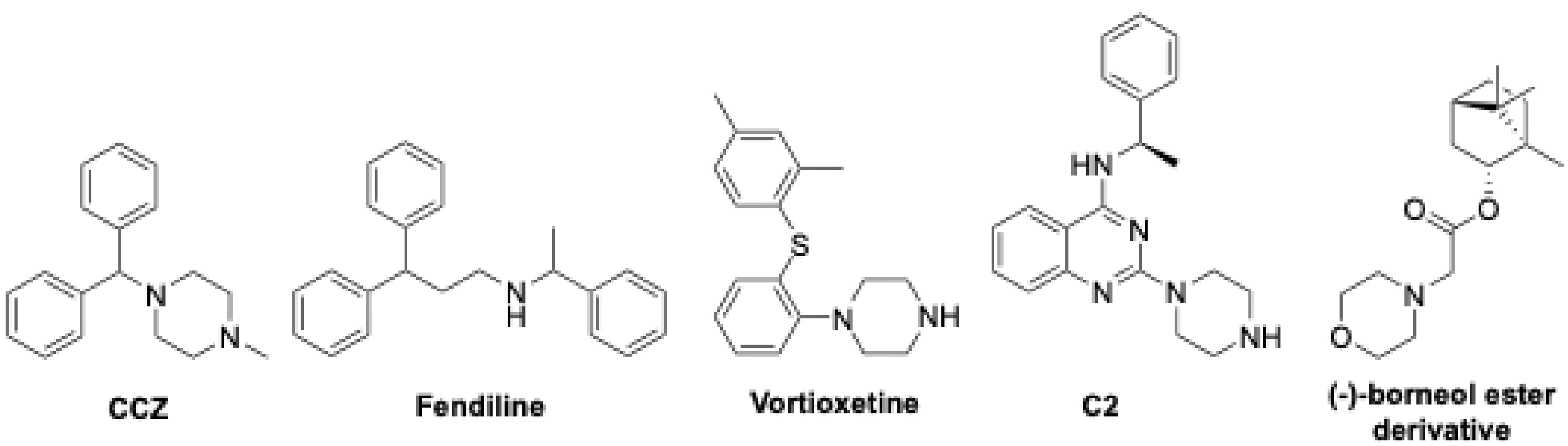

Figure S1. Representative ligands from the literature used for preliminary docking and pocket analysis to guide the pharmacophore definition (1,2)

Table S1. Selected compounds emerging from the screening.

For compounds 1, 4, 6, 8**,** 9, 10, 11 possessing stereochemical centers, the specific stereoisomer that was considered for docking is indicated.

Compound ID	Chemical Structure	MW	Commercial code
1	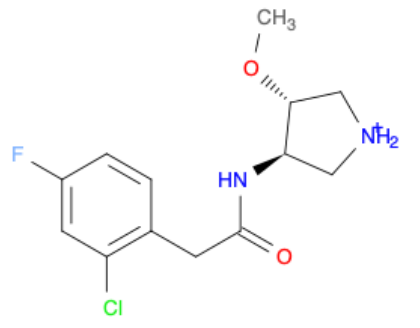	323.19	Amb31365959
2	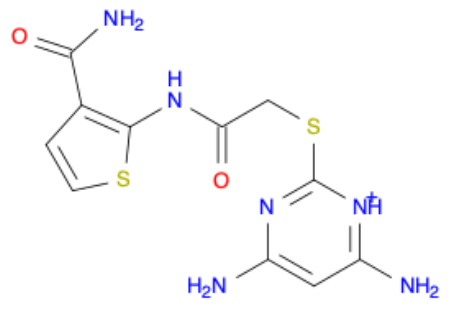	324.28	Amb520928
3	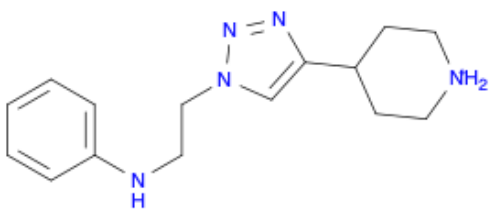	271.36	Amb22310043
4	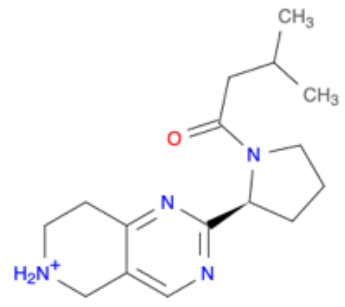	324.84	MCULE-9942300320
5	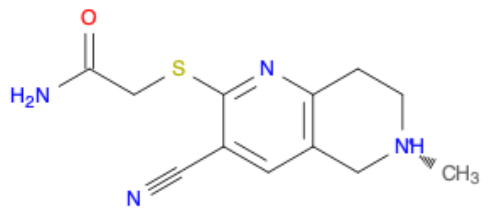	262.33	MCULE-7042633591
6	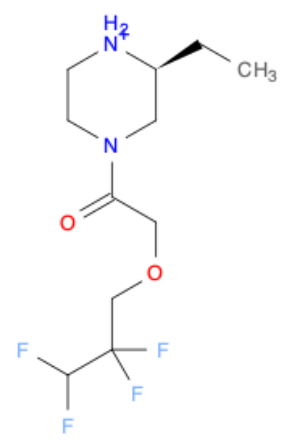	286.26	MCULE-7635943229
7	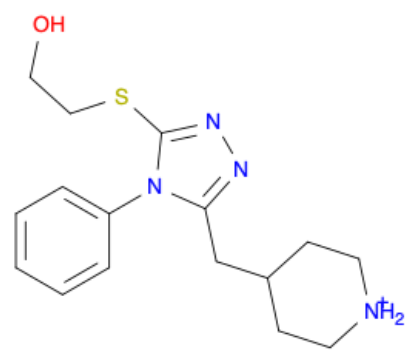	354.89	MCULE-2123355862
8	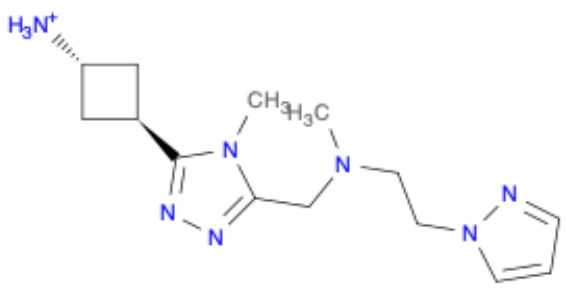	289.38	MCULE-5953273415

9	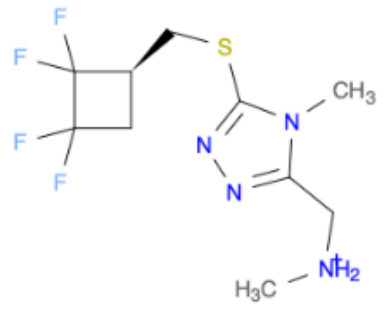	334.76	MCULE-4248285817
10	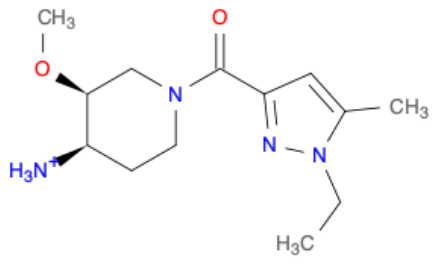	266.33	MCULE-3835849185
11	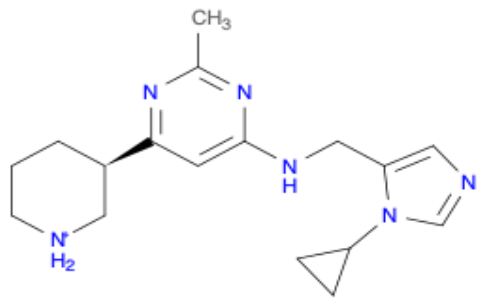	312.41	MCULE-1882300118

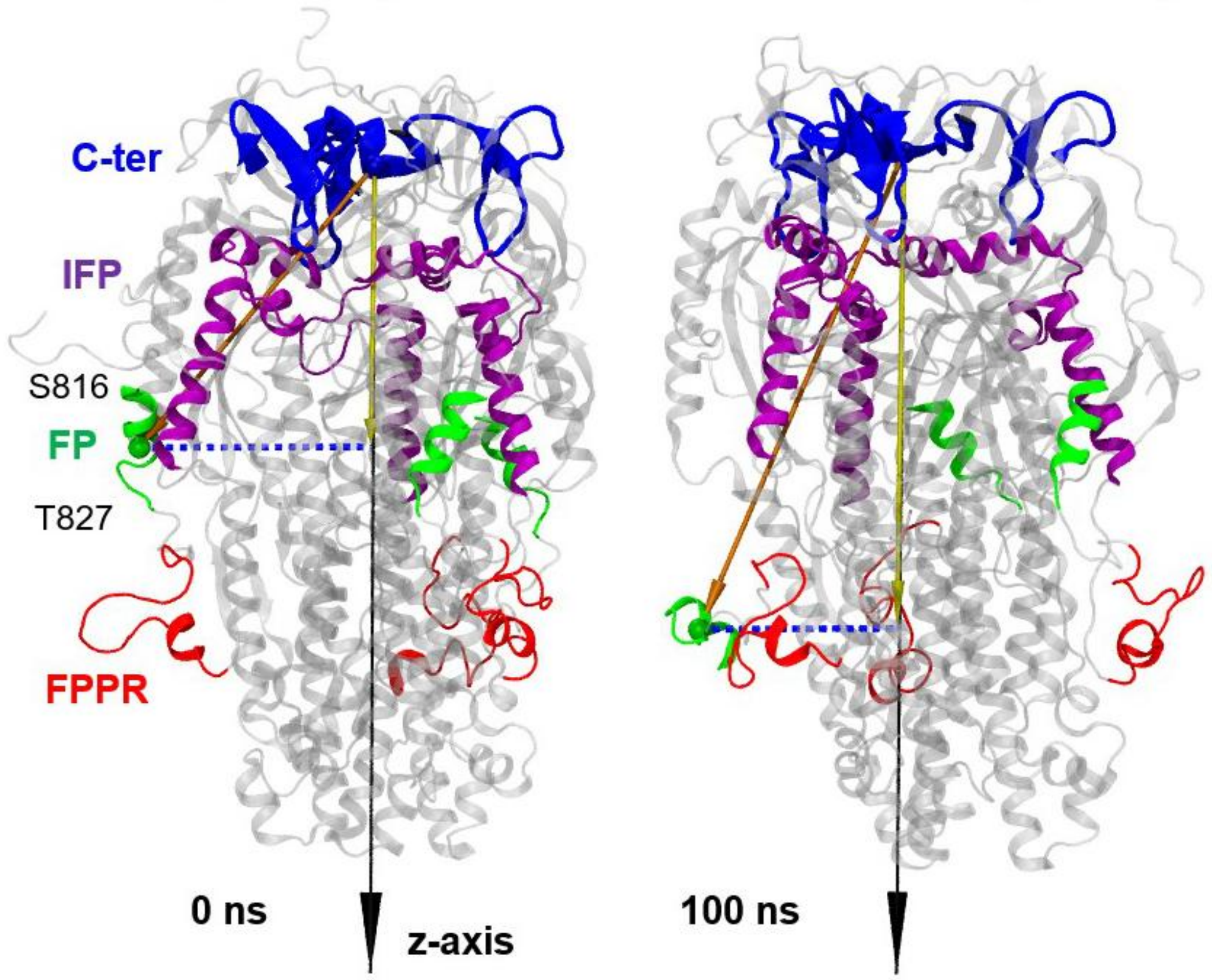

Figure S2. Representative snapshots of steered molecular dynamics (SMD) simulations showing constant-velocity pulling of the fusion peptide (FP, residues 816–834) from the prefusion Spike architecture. The FP of a single protomer was pulled along the positive z-axis away from the S2 core, mimicking exposure following S2′ cleavage by host proteases (e.g., TMPRSS2 or endosomal cathepsins). The vector connecting the FP and the C-terminal reference region is shown in orange, while the instantaneous displacement vector is shown in yellow. The dashed blue line represents the projection of this displacement along the z-axis, used to compute the mechanical work of FP extraction in both apo and ligand-bound systems. The C-terminal region (residues 1097–1122), used as a reference and restrained during SMD simulations, is shown in blue. The FP, fusion peptide proximal region (FPPR, residues 835–856), and internal fusion peptide (FPi, residues 866–909, recently recognized as the "true FP" (3) are shown in green, red, and purple, respectively. The centre of mass of the pulled FP backbone atoms is highlighted as a green sphere. All remaining protein regions are shown as a semi-transparent white representation.

Multiple sequence alignment

The multiple sequence alignment (MSA) was constructed from three predefined groups (G1–G3), representing human coronaviruses, non-human sarbecoviruses, and SARS-CoV-2 variants, respectively.

G1 — Human coronaviruses (UniProt Spike sequences)

P0DTC2   SARS-CoV-2

P59594   SARS-CoV

K9N5Q8   MERS-CoV

Q5MQD0   HCoV-HKU1

P36334   HCoV-OC43

P15423   HCoV-229E

Q6Q1S2   HCoV-NL63

G2 — Non-human sarbecoviruses (NCBI genomes)

MN996532.2   RaTG13

MZ937000.1   BANAL-20-52

MZ937001.1   BANAL-20-103

MZ937003.2   BANAL-20-236

MZ081381.1   RpYN06

MW251308.1   RacCS203

KF367457.1   WIV1

KC881005.1   RsSHC014

MZ190138.1   Khosta-2

MT040335.1   PCoV_GX-P1E

MT040334.1   PCoV_GX-P5L

MT040336.1   PCoV_GX-P5E

MT072864.1   PCoV_GX-P2V

G3 — SARS-CoV-2 variants (NCBI genomes)

MN908947.3   Wuhan-Hu-1

OL461702.1   Alpha (B.1.1.7)

OR353131.1   Beta (B.1.351)

OR322707.1   Gamma (P.1)

OR323381.1   Delta (B.1.617.2)

OR357845.1   Omicron BA.1

PP405604.1   Omicron BA.2

PP847902.1   Omicron BA.5

PP832907.1   Omicron JN.1

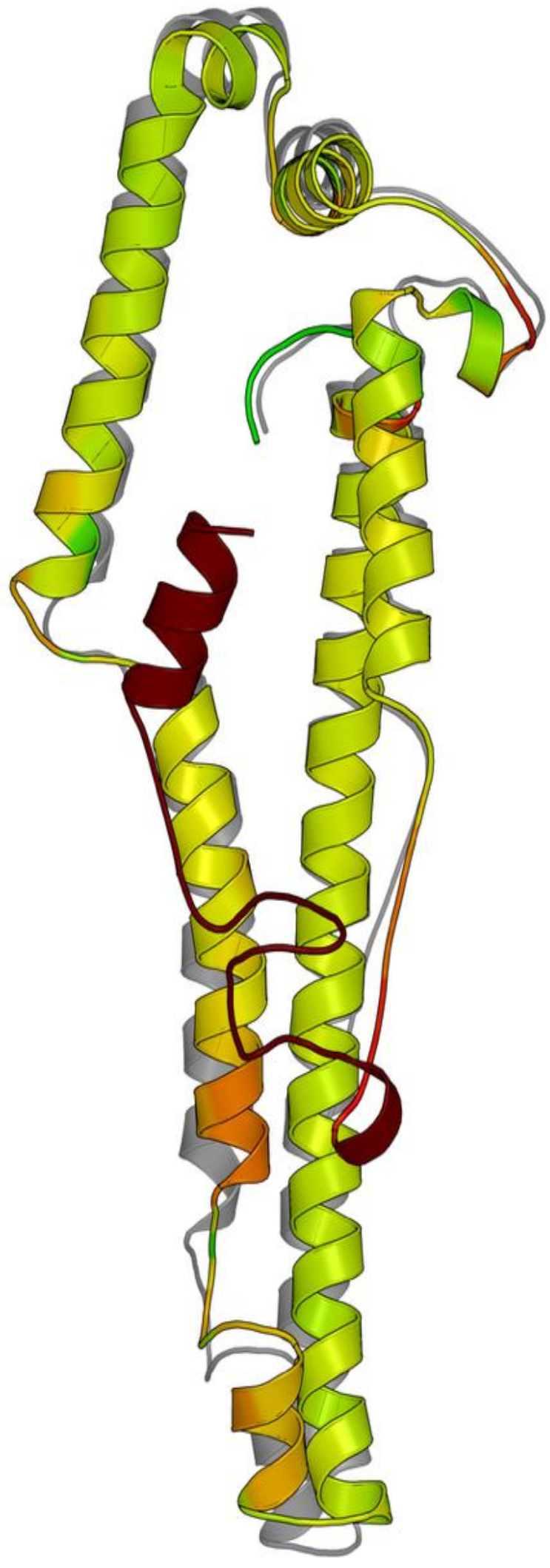

Figure S3. Structural superposition of the subset (resid 710-1149) reference spike structure (PDB 7WEC) and the modeled Omicron variant. The reference structure (chain A from 7WEC) is shown in light gray, whereas the modeled Omicron structure (chain P) is colored according to the C alpha displacement relative to the reference. Residues are mapped on a green-to-yellow-to-red scale, corresponding to increasing deviation (green = 0 Å, yellow = 1 Å, red = 2 Å). Residues 819-858 of the Omicron model, missing in 7WEC, are highlighted in dark red.

Correlation Between Shape-Fit Score and Inhibitory Activity

To evaluate whether the pharmacophore-derived shape complementarity can be associated with biological activity, a correlation analysis was performed between the shape-fit score obtained during pharmacophore screening and the experimentally determined IC_{50} values of the selected compounds. Monotonic association between shape-fit score and IC50 values was evaluated using the Spearman rank correlation coefficient. A statistically significant inverse monotonic correlation was observed between shape-fit score and IC_{50} values:

$$\rho = -0.61, \qquad p = 0.046$$

indicating that compounds with improved shape complementarity toward the consensus pharmacophore generally exhibit lower IC50 values and therefore have increased inhibitory potency.

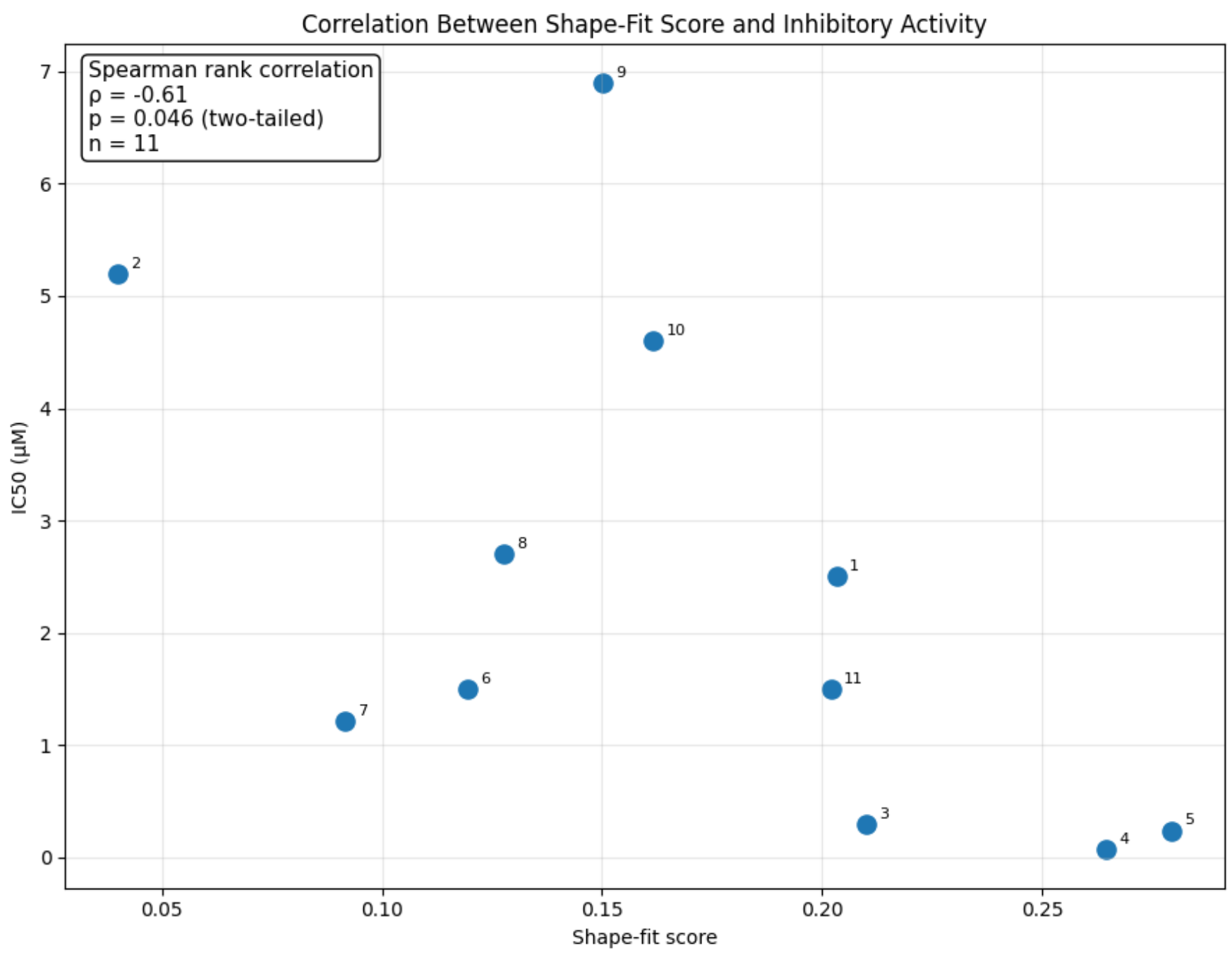

Figure S4. Scatter plot of pharmacophore-derived shape-fit score versus experimentally determined IC_{50} for compounds **1-11**. Statistical analysis was performed using the Spearman rank correlation coefficient ($\rho = -0.61$, $p = 0.046$, two-tailed, $n = 11$).

References

1- Hu X, Chen CZ, Xu M, Hu Z, Guo H, Itkin Z, Shinn P, Ivin P, Leek M, Liang TJ, Shen M, Zheng W, Hall MD. Discovery of Small Molecule Entry Inhibitors Targeting the Fusion Peptide of SARS-CoV-2 Spike Protein. ACS Med Chem Lett. 2021,12,1267-1274.

2- Yarovaya OI, Shcherbakov DN, Borisevich SS, Sokolova AS, Gureev MA, Khamitov EM, Rudometova NB, Zybkina AV, Mordvinova ED, Zaykovskaya AV, Rogachev AD, Pyankov OV, Maksyutov RA, Salakhutdinov NF. Borneol Ester Derivatives as Entry Inhibitors of a Wide Spectrum of SARS-CoV-2 Viruses. Viruses. 2022, 14,1295.

3- Shi W, Cai Y, Zhu H, Peng H, Voyer J, Rits-Volloch S, Cao H, Mayer ML, Song K, Xu C, Lu J, Zhang J, Chen B. Cryo-EM structure of SARS-CoV-2 postfusion spike in membrane. Nature. 2023, 619,403-409.
